# Advances in Influenza Virus Research: A Personal Perspective

**DOI:** 10.3390/v10120724

**Published:** 2018-12-18

**Authors:** Kanta Subbarao

**Affiliations:** 1 WHO Collaborating Centre for Reference and Research on Influenza, The Peter Doherty Institute for Infection and Immunity, Melbourne, VIC 3000, Australia; kanta.subbarao@influenzacentre.org; Tel.: +61-03-9342-9300; 2 Department of Microbiology and Immunology, The University of Melbourne, The Peter Doherty Institute for Infection and Immunity, Melbourne, VIC 3000, Australia; kanta.subbarao@unimelb.edu.au

**Keywords:** influenza, technical advances

## Abstract

Technical advances in the last decade have made it possible to investigate influenza virus infection from the cellular and subcellular level to intact animals and humans. As a result, we have gained a new understanding of the virus and disease.

## 1. Introduction

Influenza viruses and the disease that bears the same name have fascinated researchers for decades. While some researchers are interested in the clinical illness, disease severity and its complications, others focus on virus biology, specific viral proteins, and virus-host interactions whereas some immunologists have used influenza viruses and proteins as model antigens in basic research. Technical advances in the last decade, many within the last few years, have made it possible to investigate influenza virus infection from the cellular and subcellular level to intact animals and humans. As a result, we have gained a new understanding of the virus and disease; some dogma has been questioned or even overturned and, in some instances, investigators have re-discovered what was previously known. I will present my own perspective on advances that have occurred during my career, with the caveat that this is not a comprehensive or thorough assessment of progress in the field as a whole.

## 2. Ten Remarkable Advances

**1.** The technique of reverse genetics, the generation of an infectious influenza virus starting from the sequence of the virion RNA, is a very powerful technique that has changed our ability to study the biology of influenza viruses, down to the level of individual amino acid residues in specific viral proteins and has certainly changed how vaccines are or can be made. The technology evolved over a few years from the ability to rescue (or recover) one cloned viral gene segment using a helper virus to supply the remaining seven gene segments [1], to plasmid-based reverse genetics that did not require a helper virus but required co-transfection of 12 to 17 plasmids [2,3], and then to an optimized system of using eight bi-directional plasmids that can be transcribed to produce viral and messenger RNA [4]. Reverse genetics is now widely applied in all aspects of influenza research (see below).

**2**. The 1918 influenza pandemic was the largest single infectious disease event of the 20th century, causing an estimated 50 million deaths worldwide. For decades, it was assumed that the reason that the 1918 pandemic was so severe was that it was caused by a particularly virulent influenza A virus. The painstaking work to recover and sequence short overlapping RNA fragments that covered the entire influenza virus genome from paraffin-embedded formalin-fixed tissue blocks from soldiers who had died during the 1918 pandemic, confirmed by recovery of similar sequence data from the body of a fatal case of influenza that was buried in the permafrost in Alaska, revealed that the 1918 influenza A virus did not contain molecular signatures associated with highly virulent influenza viruses, such as a highly cleavable hemagglutinin protein [5,6]. The 1918 influenza virus was reconstructed by reverse genetics techniques and studied in animal models including mice, ferrets and non-human primates [7,8,9,10]. These studies revealed that the virus elicits an exaggerated and aberrant inflammatory response that is not controlled by the innate immune system [7,8]. Bacterial co-infection was also a major factor in the mortality associated with the 1918 influenza pandemic [11]. Secondary bacterial infection following influenza and its contribution to severe disease is an active topic of research.

**3.** For many years it was believed that animal influenza viruses were restricted in their ability to directly infect humans. This was based on the fact that (i) the 1957 and 1968 pandemic viruses were reassortant viruses that derived two or three gene segments from an avian influenza virus and the remaining gene segments from a previously circulating human influenza virus [12,13] and (ii) avian influenza A viruses administered to humans during experimental infection studies failed to infect or cause severe illness [14]. Several events over the last 20 years have changed this perception: first, the reconstruction of the genome of the 1918 virus indicates that the virus was a wholly avian influenza virus [15] and second, direct infection of humans by avian influenza viruses of different subtypes including H5, H6, H7, H9 and H10 have been identified, associated with illness ranging from mild (H9) to severe sporadic infections (H6 and H10) and severe illness in hundreds of sporadic cases (H5, H7) [16,17,18,19,20,21].

**4.** The identification of influenza infections at the animal human interface since 1997, caused by many different influenza A subtypes, has led to closer collaboration and communication between human and animal public health researchers, surveillance in wild birds and poultry and greater recognition of the ‘One Health’ vision [22].

**5**. As a result of advances and access to molecular methods and international sharing of genetic sequence data, the sequences of H7N9 avian influenza viruses that caused human and poultry infections in China have been available for analysis by researchers around the world.

**6**. Advances in antiviral drug development: Amantadine and its derivative Rimantadine are ion-channel inhibitors that were available for use for prophylaxis or treatment of seasonal human influenza till the early 2000s, when resistance to these drugs emerged and spread globally [23]. Fortunately, the neuraminidase inhibitor class of drugs, that were generated by rational computer-aided design [24] remain effective and are now the mainstay for treatment. In 2018, nearly 20 years after the licensure of the neuraminidase inhibitors, a polymerase inhibitor has been licensed for treatment of influenza in Japan and the USA [25].

**7.** Immortalization of B cells and characterization of the antibody repertoire using phage display libraries [26,27,28] following H5N1 infections and more widely, following the 2009 H1N1 pandemic [29,30], facilitated the identification of a highly conserved epitope in the HA stem. A similar murine antibody was first described in 1993 [31]; the conserved HA stem epitope was re-discovered using the equivalent human antibodies [32]. The HA stem is one of the most promising targets for the development of a universal influenza vaccine.

**8.** With the application of molecular techniques [33] and B cell probes [34], a much more detailed understanding of the antibody response to the haemagglutinin and neuraminidase [35] of influenza viruses is emerging, that includes mapping of epitope specificity and characterization of the role of glycans in shielding epitopes [36,37,38].

**9.** Following a long gap after the first report of the crystal structure of the influenza A (H3) HA in 1981 [39], there has been an explosion of structural information in the last decade of different HA subtypes, many in association with well-characterized antibodies [32,40,41,42]. In conjunction with advances in protein engineering, this knowledge will enable rational design of vaccines [43,44]. The conventional inactivated influenza vaccine may be replaced in the future by a vaccine containing a computationally designed and optimized or antigenically advanced HAs [45,46], presented as nucleic acid [47,48,49] or protein.

**10.** Some of the most remarkable advances in our understanding of the biology of influenza viruses, the pathophysiology of infection at the level of the single cell as well as intact animals and our understanding of virus-host interactions have resulted from the application of advances in imaging techniques. It is possible to track and image infection in single cells and in intact animals [50,51], to study how gene segments interact as they are transported through the cytoplasm and assemble into daughter virions [52], to determine how many gene segments are present in a virion that can cause a productive infection [53] and to study reassortment and packaging of influenza virus gene segments [54]. Labelled viruses have been used to identify which cells are infected in an intact animal following intranasal administration of live virus, to explore whether viral replication in vivo is uniform [55] and what happens to bystander cells, and which cells survive infection in vivo [56,57] and what they can tell us [58].

Despite the many advances, several challenges remain. The controversies regarding dual-use research of concern (DURC) and the risks and benefits of conducting and sharing information gleaned from such research have been polarizing. The resulting regulations pose an ongoing challenge for individual investigators, institutions, journal editors and funding bodies. Although the prospects of improved and/or novel influenza vaccines are good, equal access to vaccines and antiviral drugs for the global population remains a challenge.

Many big questions about influenza are yet to be answered. Why is influenza a seasonal disease in temperate climates? What makes some influenza viruses more virulent than others? Why do some influenza viruses induce a cytokine storm? What makes some influenza viruses transmissible in people while others are not? Will it be possible to forecast influenza evolution? What is the mechanistic basis for the concept of immunologic imprinting [59]? Will a universal influenza vaccine become a reality and will it be an adjunct to or replace conventional influenza vaccines? I am confident that the advances in molecular and quantitative virology, immunology, evolutionary biology and ecology, transmission and environmental control, modelling and novel therapeutics will answer many of these questions. With the tools that are available and a very talented group of young scientists who have entered the field, the future of influenza research is bright!
